# Intellectual humility and the learning sciences: can self-reports and behavioral measures coexist to understand civic engagement?

**DOI:** 10.3389/fpsyg.2024.1451306

**Published:** 2024-10-14

**Authors:** Matthew Lira, Stacey McElroy-Heltzel

**Affiliations:** Learning Sciences and Educational Psychology, Department of Psychological & Quantitative Foundations, College of Education, The University of Iowa, Iowa City, IA, United States

**Keywords:** intellectual humility, learning, politics, EDA, affect, epistemology, multi-modal learning analytics

## Abstract

Recent political events across the globe have illustrated a resurgence in people’s intolerance to ideas different from their own. We mobilize the idea of intellectual humility to assess how extant psychological theories account for individual differences in people’s tolerance for conflicting ideas. Then, we introduce concepts from the Learning Sciences to determine how alternative methodologies could augment research on intellectual humility and civic engagement. Last, we summarize these analyses by pointing to their relations with three intersecting challenges and solutions regarding studying IH in multiple contexts and with new multiple data sources.

## Introduction

Political polarization in the United States has increased in the past two decades (Pew Research Center, 2022). Some studies have indicated that such polarization fuels dehumanization of political opponents, which can threaten the foundation of democratic processes (Kubin et al., 2023). Intellectual humility (IH), a character virtue that involves owning one’s knowledge limitations (Haggard et al., 2018), is proposed to mitigate the cognitive biases associated with polarization (Bowes et al., 2020). Studies have indicated that IH in politics is related to less affective polarization and more responsiveness to information representing an opposing viewpoint (Bowes et al., 2020; Krumrei-Mancuso and Newman, 2020).

Alternatively, a politician engaging in humble communication—especially when paired with a negative facial expression (e.g., sad)—elicits negative evaluations (D’Errico, 2019). This however shifts toward positive evaluations when a female rather than a male politician presents humble communication (D’Errico et al., 2022). These findings motivate the need to understand the context-specificity of IH within the political domain. For example, what is not clear from these two studies is whether an individual’s own level of IH moderates their evaluation of politicians. Would an individual higher in IH be more likely to support politicians with less extremist views?

**Figure 1:**
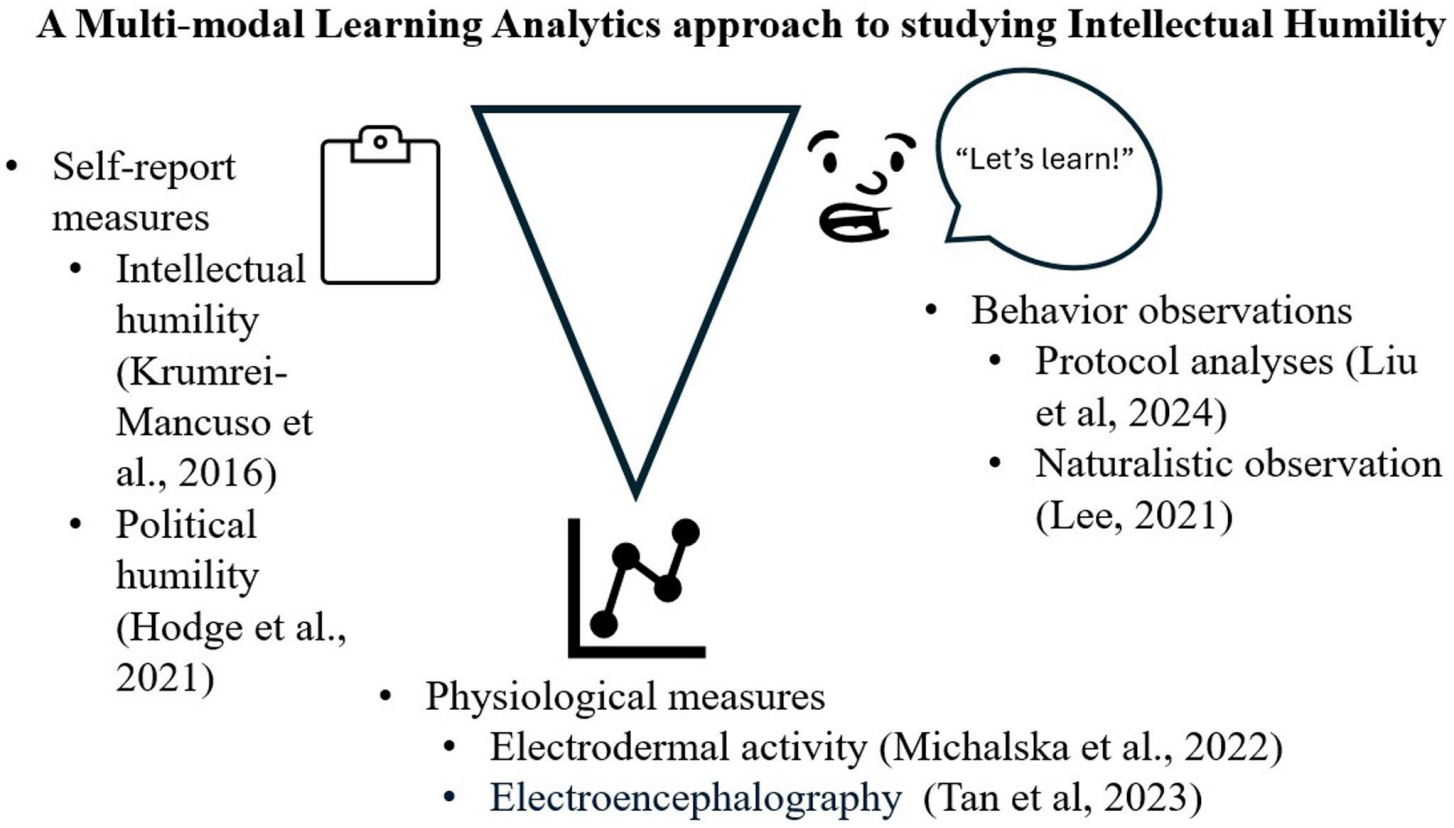
The Tri-partite Model of Emotion as assessed with MMLA methods. Representative investigations provided as examples for self-report (left), overt affective displays (right), and covert physiological arousal (bottom).

The Tri-partite Model of Emotion offers a research paradigm that situates IH in context. This model predicts that (a) self-report of subjective mental states, (b) affective displays, and (c) autonomic physiological arousal coordinate (Michalska et al., 2022). If a person says, “I feel curious during political debates,” they should also show display positive affect and be physiologically activated. Prior work partially supports this hypothesis; students who reported finding an engineering activity engaging also displayed greater physiological arousal, but not necessarily overt positive affect (Lee, 2021). Investigations that assess IH in social contexts can supplement our understanding of how IH manifests and how to cultivate in critical domains such as politics. In this perspective piece, we describe how methodologies from the Learning Sciences (LS) can augment methods typically used to study IH in the discipline of Positive Psychology (Figure 1).

## Empirical approaches to IH raise context-specificity questions

Scholars have noted two measurement concerns that need to be addressed in order to appropriately interpret and apply empirical findings to real-world contexts. These include (1) whether an individual can accurately self-report their own IH (McElroy et al., 2014) and (2) the need to contextualize measurement (Porter et al., 2022). An individual higher in IH should recognize the human potential to have blind spots, including in their own ability to perfectly know where their intellectual limitations lie. They may accurately assess themselves as high, but not perfect, in IH (e.g., a score of 4 on a scale of 1–5), while the arrogant individual may be more prone to inaccurately rate themselves as perfect in IH (e.g., a score of 5 on a scale of 1–5). To address the first concern, early measures of IH were developed as informant reports (e.g., McElroy et al., 2014).

However, informant reports are not inherently sensitive to context and cannot capture an individual’s internal cognitive processes. Indeed, self and informant reports appear to capture different aspects of IH. For example, self- and informant reported IH have demonstrated differential relationships with other constructs in the nomological net of IH (Meagher et al., 2021). Although the reliability and validity of self-reported IH remains an outstanding empirical question, self-reports remain the most pragmatic and accurate means of assessing an individual’s *perception* of their own cognitive processes. While scholars disagree on the extent to which IH is interpersonal, most all agree that the theorized cognitive features of IH are essential (Porter et al., 2022).

Second, IH is thought to manifest and be most apparent under strain (Davis et al., 2011), highlighting the need to attend to context. If an individual is not being challenged to attend to their intellectual limitations, they lack the appropriate opportunity to demonstrate ownership of those limitations. They might simply feel indifferent, or even have a tendency of over-owning their limitations regardless of context (i.e., display intellectual servility; Battaly, 2021). For example, an individual might be very committed to their stance on abortion (an opportunity to display IH instead of arrogance) but feel indifferent about immigration. This represents the problem of noise in general IH measures because we do not know the specific context individuals are calling to mind when responding to these measures. Furthermore, if an individual holds expertise in a topic (e.g., climate science), deferring to someone without similar expertise would not be a virtuous display of IH, but rather display the vice of intellectual servility (Battaly, 2021).

For example, some measures of domain-specific IH have been utilized in experimental studies that target a behavior, but these measures are also associated with belief commitment. Individuals with low to moderate commitment to their beliefs had more IH about those beliefs than individuals high in their belief commitment (Hopkin et al., 2014; Hoyle et al., 2016). However, if IH only manifests in contexts where individuals are not strongly committed to their beliefs, its theorized role in mitigating issues of bias and polarization is markedly limited. These findings also suggest that emotion might regulate individuals’ epistemic and moral stances (McLaughlin et al., 2023). While Van Tongeren et al. (2023) present some compelling ideas for behaviorally assessing IH, one component that remains missing are IH self-reports related to people’s experienced emotions.

To address the limitations in assessing IH solely via self-report, we draw upon the history of the Learning Sciences (LS) to characterize methodological problems and identify potential solutions to Positive Psychology’s investigations into IH. LS is a multi-disciplinary field that studies human learning in context (e.g., online, classrooms, museums, and synagogues) and across domains—i.e., chemistry and history (Herrenkohl and Polman, 2018; Hoadley, 2018). In undertaking this effort, we do not argue that scholars abandon self-report measures per the Tripartite Model of Emotion. Rather, we aim to identify complementary methods for traingulating IH. To accomplish this, we will use a historical case concerning students’ personal epistemology to build guideposts for considering context, and then describe how multi-modal learning analytics approaches could augment our understanding of IH in context.

## Methods from the learning sciences assess constructs with context-specificity

LS emerged from cognitive science of the 1980s to address learning in real-world contexts (Hoadley, 2018; Kolodner, 2004). This effort demanded relinquishing controlled studies and standardized measures to embrace qualitative methods that delivered ecological validity. This culminated in a broad learning theory that emphasized context—*situated cognition* (Brown et al., 1989). Situated perspectives argue that people’s knowledge is tethered to the social and material context. This view contrasts with information processing models of knowledge (Newell and Simon, 1972) or trait-theory perspectives that assume relative stability (Anastasi, 1983).

Situated perspectives predict that IH will manifest to different degrees across varied social, material, and intellectual contexts. At present, we have limited evidence that people’s IH varies with context (see Zachry et al., 2018), but a related construct—*personal epistemology*—offers an illustrative case that could guide investigations. Epistemology is the branch of philosophy concerned with the origins, nature, and limits of knowledge (Chinn et al., 2011). Personal epistemology refers to people’s beliefs about learning and knowledge—its source and certainty (Hammer and Elby, 2003; Hofer, 2004). LS scholars shifted from modeling personal epistemologies as context-insensitive to context-sensitive. This shift offers guideposts for future IH studies.

### Stable personal epistemologies

Seminal investigations into university students’ developing personal epistemologies revealed stable and predictable patterns that transition from non-normative to normative scientific beliefs about knowledge (Perry, 1970). Although different models exist, students generally transition between three distinct epistemic phases during their undergraduate years (Weinstock, 2006). First, *absolutist* involves believing one true account on a controversy. Second, *multiplicist* involves believing in many accounts on a controversy irrespective of correctness. Third, *evaluativist* involves constructing an account on a controversy based upon weighing evidence.

The evaluativist stage bears the closest relation to IH. We conjecture that higher IH people could evaluate evidence for or against their own knowledge limits better than lower IH people. The evaluativist stage reveals an increased sensitivity to context and a sensitivity to judging knowledge as more or less veridical depending upon multiple dimensions (e.g., expertise, reliable practices). Early work, however, cataloged students’ stable epistemologies to demonstrate that students varied in their espousing formal stances, such as the tentativeness of knowledge or uncertainty (Hofer, 2004). Perry (1970) and later others (Carey and Smith, 1993; Driver et al., 1996) illuminated a stable, stage-like trend—students first see knowledge as certain (i.e., absolutist) and determined by authority, then later as pluralistic with everyone having a right to an opinion (i.e., multiplicist), and last as relative or context-dependent (i.e., evaluativist). Stage-theoretical perspectives predict that IH people should attend to context always, e.g., evaluating *physics* claims across macroscopic *or* sub-atomic scales, *economic* claims across first world *or* developing nations, and so forth.

Thus, this stage-like model of epistemic growth tacitly assumes invariance. Therefore, it offers minimal insight into mechanisms for transitioning between epistemologies, i.e., learning. We contend that it similarly offers little insight into how IH develops or how to cultivate it. To determine learning mechanisms scholars need to attend to social and material factors that induce shifts in people’s professed epistemology. An emerging research line illustrated that people’s epistemologies varied with context. Video data on students’ contextualized actions ushered in a dynamic epistemology view. The discrepancy between a student’s verbal expression and action directs IH studies toward video data and other emerging LS methods.

### Dynamic personal epistemologies

As theories and methods (e.g., video analysis) for understanding students’ personal epistemologies matured in LS, a resolution emerged that students shift dynamically between epistemic stances. For example, biology students shift in how they view the nature of biological knowledge within minutes (Watkins and Elby, 2013). Moreover, the idea of a stable personal epistemology proved problematic. Russ (2014) critiqued the very notion of *an* epistemology *of* science—pointing out that science is a social construct. Therefore, students must recognize a context as “science” or not if they are to “activate” their alleged science epistemology as distinct from all other reasoning. This seems implausible given that situations overlap (e.g., politicized science like stem-cell research). As multi-dimensional contextual factors change, people become more or less likely to cue different epistemic beliefs (Hammer and Elby, 2003).

Dynamic perspectives predict that IH people will own their intellectual limitations dependent upon social, material, and intellectual contexts that induce people to shift between humble, arrogant, servile, or prideful stances. This perspective emerged from observing students’ actions. New theory and methods distinguished between people’s *professed beliefs* as expressed in de-contextualized scenarios and people’s *enacted beliefs* in context (Sandoval, 2005). Students’ enacted beliefs mediate the relation between their professed beliefs and their performance on science assessments (Lang et al., 2021). Likewise, measures of general and domain-specific topics reveal that students’ demonstrate variable levels of epistemic cognition by context domain (Barzilai and Weinstock, 2015)—e.g., students accept uncertainty in *history* more so than in *biology* despite being taught that science involves uncertainty. Moreover, experimental approaches demonstrated that failure to situate science problems in context eliminates the effectiveness of epistemic interventions (Klopp and Stark, 2022).

Emerging methods in LS revealed further discrepancies between students’ professed and enacted beliefs. For example, Sandoval (2005) documented a history of investigations that illuminated a distinction between *formal* epistemology and *practical* epistemology. Practical epistemology refers to students’ beliefs about their own learning that guides their actions in school. Whereas students will memorize de-contextualized epistemic stances that they are taught to recite in school, their practical epistemologies may remain unchanged. Students might appear an absolutist who appeals to authority at one moment, but an evaluativist who considers context at another. It makes sense to trust your doctor’s authority when deciding to take medicine, but not when the same doctor testifies on behalf of a drug company that pays them.

Such shifts reveal context-sensitive epistemic cognition rather than a stable naïve epistemology. We conjecture that people will manifest IH dynamically if their actions are assessed in different contexts. Students’ formal and practical reasoning from moment–moment demonstrates that students consider sources in ways that vary with context. Hammer and Elby (2003) documented students’ shifts between seeing knowledge as constructed from scientific methods *and* as propagated by authority across contexts. Stable views on personal epistemology would have emphasized that students held an absolutist view on knowledge and that instructors needed to shift students toward the evaluativist view. But Hammer and Elby demonstrated that people hold epistemic resources that operate as useful in some contexts but not others. Sometimes students need to trust the periodic table to accomplish other work (cf. Russ, 2014). This is not a naïve epistemic stance—it is a practical one.

Recent investigations amenable to the Tripartite Model reveal that previously ignored contextual factors, such as the science students’ emotional state, interact with their epistemic engagement (Jaber and Hammer, 2016a, 2016b). Emotion further destabilizes stage-like models of epistemic growth in ways that motivate new methods for assessing personal epistemologies and IH. Because emotion is inherently dynamic, it stands to reason that IH individuals may nevertheless display variable IH with changes in their emotions when learning in politics, science, or related domains. Although methods from personal epistemology have historically ignored emotion, the emerging field of Multi-Modal Learning Analytics offers new tools for assessing how IH manifests in physiological measures.

### LS methods’ utility for assessing IH

First, we propose that scholars should retain IH self-report measures. These measures assess subjective mental states critical to testing the Tripartite Model of Emotion and theories of IH. We suggest, however, validating self-report with behavioral observations. Two broad approaches would address IH validity concerns. First, use the contextualized approach from epistemic cognition to assess *enacted IH*. In contrast to professed epistemology, peoples’ enacted epistemology requires people to use knowledge to guide action. We could design tasks that require people to act with IH in situations that demand their owning their intellectual limitations. These tasks could occur in laboratory settings, classrooms, or professional or civic contexts.

Second, we propose borrowing from Multi-Modal Learning Analytics (MMLA). We argued that IH cannot involve indifference and is most apparent under strain. MMLA uses markers such as affective displays and delivers physiological measures like electrodermal activity (EDA) (Sharma and Giannakos, 2020). EDA measures skin conductance to assess autonomic nervous system arousal and affective engagement (Lee, 2021), providing insight into the temporal dynamics of strain during learning, distinguishing IH from indifference. Although there are not established markers for IH using EDA data, we hypothesize that individuals who are indifferent would display less physiological reactivity compared to IH and arrogant individuals. Because EDA measures lack gold standards for collecting and analyzing these data (Horvers et al., 2021), we propose coordinating EDA with video data to guide interpretation. To summarize, we hypothesize a profile whereby individuals higher in IH would (1) display moderate physiological reactivity as measured by EDA; (2) self-report moderate to high levels of IH; and (3) display IH congruent behaviors within a specific context.

We previously employed this strategy (Liu et al., 2024). Borrowing from the heuristics and biases literature (Kahneman, 2011), we used an established problem called the 2, 4, 6 task to induce the confirmation bias (Wason, 1960). This allowed us to conduct a protocol analysis that identified the sequence of cognitive, affective, and behavioral engagement markers participants displayed (Ericsson and Simon, 1993). We then completed a contrasting case analysis illustrating that one individual high in self-reported IH displayed sustained affective engagement (e.g., smiling), while the low IH individual displayed alternating periods of negative affect (e.g., grimacing). Moreover, the high IH individual displayed lower reactivity than the low IH individual, as measured by EDA variability (Li and Lund, 2012). These initial results deliver insight into how people’s IH manifest across the three levels of the Tripartite Model and in context.

## Discussion

IH offers value for theorizing about how to cultivate people’s ability to own their intellectual limitations. To cultivate IH, we must understand how to measure it in context. We argued that extant IH measures raise two concerns (1) the validity of self-report and (2) the stability of IH across contexts. The Learning Sciences responds to the first concern by distinguishing between people’s professed and enacted epistemic beliefs—this distinction motivates overt behavioral observations that determine how people with different professed IH enact IH. We proposed that MMLA (e.g., EDA) addresses one aspect of the second concern—namely, that EDA measures detect heightened arousal and thus eliminate invalid displays of IH that involve indifference. This proposal aligns with the Tripartite Model as a guide for designing future studies.

EDA measures, however, leave open the possibility that IH individuals manifest better emotion regulation than non-IH persons. Different cognitive, affective, and behavioral engagement nevertheless reveals how IH manifests across contexts in people’s strategies, emotional displays, and sustained problem solving. MMLA offers tools for assessing how IH manifests covertly as when a person experiences heightened stress levels during learning. Although MMLA (e.g., EDA and video data) pose serious time and financial challenges, empirical investigations that coordinate these data with self-report measures ensure the predictive validity of IH *in situ* (Lee, 2021; Liu et al., 2024).

In relation to the IH in the context of civic engagement, maintaining democracies means that societies must decide whether individuals hold the intellectual faculties to cope with political polarization. We maintain the hope that people can display IH and that we can cultivate it by understanding its manifestation in context. Educating for IH reflects a promising possibility if only because of its current necessity. Contemporary societal challenges demand multiple expert viewpoints where no individual holds the solution (e.g., climate change). To sustain our learning and engagement in such uncertain contexts will demand IH.

The efforts to cultivate IH will necessitate clarity regarding optimal measurement and whether and when IH is virtuous. We propose addressing these concerns through implementing methodological diversity to validate IH self-report measures. As the case of personal epistemology illustrated, when self-report is the only measure used, results can buttress a stable trait-like or stage-like theory. Moment-to-moment behavioral observations reveal dynamic context-sensitive variations—these measures support theorizing about learning mechanisms (Lira and Gardner, 2020; Sherin et al., 2012). Regarding the virtuous display of IH, if IH supports acquiring domain-expertise, then high self-report should support learning. Perhaps, however, IH develops only after a person learns in a domain (Kruger and Dunning, 1999).

Precedent exists for interdisciplinary IH studies (Davis et al., 2023); adding LS perspectives and methods seems suitable for IH given that they both interface with how individuals acquire knowledge. LS has well-established methodologies for behavioral and qualitative assessment in the context of learning—this may be an efficient starting point for this new frontier of IH research. Worthington and Garrett (2023) argue that, “the social context is an essential component of IH and arguably more important that ‘private mental states’” (p. 285). This point highlights the need to situate our understanding of IH to specific, relevant contexts. As it pertains to politics and civic virtue, this understanding of IH as having both domain-specific cognitive features *as well as* interpersonal manifestations better positions it as a civic virtue poised to address the political polarization currently observed in nations around the world.
